# Relevance of leadership regarding patient safety in the current context

**DOI:** 10.1590/1518-8345.0000.3484

**Published:** 2021-11-08

**Authors:** Andrea Bernardes, Carmen Silvia Gabriel, Wilza Carla Spiri

**Affiliations:** 1 Universidade de São Paulo, Escola de Enfermagem de Ribeirão Preto, PAHO/WHO Collaborating Centre for Nursing Research Development, Ribeirão Preto, SP, Brazil.; 2 Universidade Estadual Paulista Júlio de Mesquita Filho - Campus de Botucatu, Faculdade Medicina, Departamento de Enfermagem, Botucatu, SP, Brazil.

**Figure:**
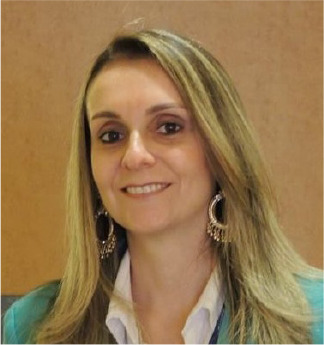


**Figure:**
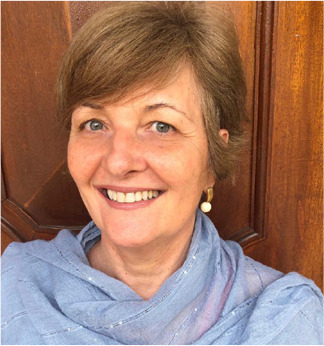


**Figure:**
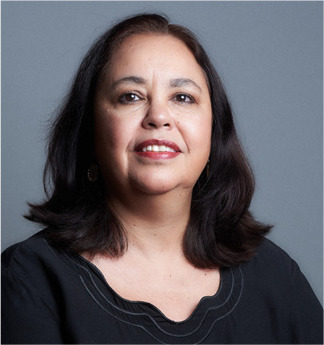


The COVID-19 pandemic is one of the most discussed issues worldwide, given the social, economic and behavioral impact generated, in addition to the high morbidity and lethality rates associated with the difficulty in early initiating the therapeutic measures, the seriousness of the cases and the overcrowding of the health services, resulting in work overload for the professionals, the increased demand for equipment and materials and the need for a sudden change in the care processes.

All these factors certainly compromise patient safety, generating coping demands by all the actors involved, with emphasis on the leaders of health systems and services. It is the leaders that must lead the process, assuming the major challenge of ensuring patient safety in this epidemiological context of facing the pandemic.

It is worth highlighting a new model that emphasizes strategies to increase safety, which reinforces that leaders must stop focusing only on searching for errors, the so-called safety 1 model, but start emphasizing the search for correct actions, as well as the way in which these are reproduced, the so-called safety 2 model^(1)^. The health system is complex and characterized by random actions, as well as by changes in the context and the conditions^(1)^, as the example of what is being currently experienced with the COVID-19 pandemic, which devastates the world with millions of lost lives. Thus, the figure of leaders who can capture the interconnections and correct actions related to safety becomes essential, so that they can deal with the complexity and dynamism of health.

In this perspective, it is evident that the leaders’ challenge is greater today, given that they need to improve on the new paradigms, seeking to understand the complexity of the system and lead the team looking at the positive aspects of patient safety, without discarding the analysis of the causes that lead to errors.

Such leaders also need to be resilient in adapting to pressure to provide safe and effective care to the patients, as well as protect the workers who experience significant emotional distress generated by the pandemic, which can adversely impact on patient safety^(2)^. It should be considered that the Nursing team is exposed to COVID-19, as it represents health professionals who are on the front line of care; thus, their safety needs to be guaranteed to ensure quality and minimization of errors. Based on encouragement from the leaders, they must report all the day-to-day events, not only the adverse results; in this way, relevant and often underused information is obtained.

A study carried out in 71 hospitals from Pennsylvania, which provide care to patients diagnosed with COVID-19, identified that 1% of the adverse events were serious, even leading to death. The others, 99%, were classified as incidents that occurred mainly in the Emergency Departments, followed by the Medical-Surgical Clinical Units and Intensive Care Units^(3)^, which can progressively compromise people’s health. Confronting this reality, for example, would mean examining all the work performed and the need for adjustments and adaptations based on the errors and correct actions that occurred.

Thus, it is observed that research agendas must continue to advance in addressing operationally relevant issues about patient safety, and new knowledge must be translated and effectively implemented in the practice at all levels, from leadership to direct and family care providers with collective and participatory involvement, with the need to consider tactics for the leaders to better balance the various competing priorities, ensuring that safety is seen and treated as a core value^(4)^.

Given the concern with the pandemic and the inevitability of quick decision-making in an often chaotic scenario, it became essential to review the care practices, invest in the health team’s educational process and in dialogic communication, as well as to improve the work process. Through investment in these areas, it is possible to strengthen the leaderships which therefore need to encourage strategies that enable engagement, appreciation and participation of the team in organizational decision-making^(5)^, contributing to successful care results.

It is concluded that changes in the work processes, as well as communication and decision-making facilitation, are essential, although they represent challenges for leaders who have joined efforts to fight against the pandemic. Reducing the number of harmful adverse events must be one of the goals of leadership and interdisciplinary teams. Thus, it is imperious to develop organizational models, capable of providing ways to ensure the safety of patients and teams alike, especially considering the current epidemiological situation.
